# EDAI Framework for Integrating Equity, Diversity, and Inclusion Throughout the Lifecycle of AI to Improve Health and Oral Health Care: Qualitative Study

**DOI:** 10.2196/63356

**Published:** 2024-11-15

**Authors:** Samira Abbasgholizadeh Rahimi, Richa Shrivastava, Anita Brown-Johnson, Pascale Caidor, Claire Davies, Amal Idrissi Janati, Pascaline Kengne Talla, Sreenath Madathil, Bettina M Willie, Elham Emami

**Affiliations:** 1 Department of Family Medicine McGill University Montreal, QC Canada; 2 Faculty of Dental Medicine and Oral Health Sciences McGill University Montreal, QC Canada; 3 Mila-Quebec AI Institute Montreal, QC Canada; 4 Lady Davis Institute for Medical Research Jewish General Hospital Montreal, QC Canada; 5 Postgraduate Institute of Medical Education and Research Chandigarh India; 6 Department of Communication Université de Montréal Montreal, QC Canada; 7 Department of Mechanical and Materials Engineering Queen’s University Kingston, ON Canada; 8 The Research Institute of the McGill University Health Centre Montreal, QC Canada; 9 Research Centre Shriners Hospital for Children-Canada Montreal, QC Canada

**Keywords:** equity, diversity, and inclusion, EDI, health care, oral health care, machine learning, artificial intelligence, AI

## Abstract

**Background:**

Recent studies have identified significant gaps in equity, diversity, and inclusion (EDI) considerations within the lifecycle of artificial intelligence (AI), spanning from data collection and problem definition to implementation stages. Despite the recognized need for integrating EDI principles, there is currently no existing guideline or framework to support this integration in the AI lifecycle.

**Objective:**

This study aimed to address this gap by identifying EDI principles and indicators to be integrated into the AI lifecycle. The goal was to develop a comprehensive guiding framework to guide the development and implementation of future AI systems.

**Methods:**

This study was conducted in 3 phases. In phase 1, a comprehensive systematic scoping review explored how EDI principles have been integrated into AI in health and oral health care settings. In phase 2, a multidisciplinary team was established, and two 2-day, in-person international workshops with over 60 representatives from diverse backgrounds, expertise, and communities were conducted. The workshops included plenary presentations, round table discussions, and focused group discussions. In phase 3, based on the workshops’ insights, the EDAI framework was developed and refined through iterative feedback from participants. The results of the initial systematic scoping review have been published separately, and this paper focuses on subsequent phases of the project, which is related to framework development.

**Results:**

In this study, we developed the EDAI framework, a comprehensive guideline that integrates EDI principles and indicators throughout the entire AI lifecycle. This framework addresses existing gaps at various stages, from data collection to implementation, and focuses on individual, organizational, and systemic levels. Additionally, we identified both the facilitators and barriers to integrating EDI within the AI lifecycle in health and oral health care.

**Conclusions:**

The developed EDAI framework provides a comprehensive, actionable guideline for integrating EDI principles into AI development and deployment. By facilitating the systematic incorporation of these principles, the framework supports the creation and implementation of AI systems that are not only technologically advanced but also sensitive to EDI principles.

## Introduction

Artificial intelligence (AI) is a branch of engineering and computer science that creates systems capable of performing tasks that typically require human intelligence such as visual perception, speech recognition, and language translation, while also analyzing complex datasets to identify patterns, make predictions, and solve problems across various domains [1-3]. AI technologies are increasingly being applied in all sectors of our society including health care systems [4-8]. These systems could be helpful in improving the effectiveness of health care delivery and facilitate achieving quadruple aims of care, that is, improved patient experience, better outcomes, lower costs, and improved clinical experience [9]. The introduction of AI in medicine and dentistry has shown promising potential [8,10,11] in screening, detection, and treatment of diseases [12,13]. AI enhances the use of electronic health records, supports clinical decision-making, and enables the development of sophisticated robots and devices to enhance care delivery and alleviate workload in health care systems [8,14].

The deployment of AI across various sectors has underscored the importance of ethical governance and the integration of equity, diversity, and inclusion (EDI) principles. Drawing on prior research from various industries, it is evident that AI technologies can both mitigate and exacerbate existing social biases, depending on their design and implementation [6,15,16]. This dual potential of AI, as highlighted in studies like Obermeyer et al [17], emphasizes the necessity for incorporating EDI principles from the onset of development to ensure technologies promote inclusivity and prevent social inequality. Moreover, research on algorithmic accountability underscores the need for transparency and explainability in AI systems, requiring designs that provide traceability of decisions and clear data use to comply with international regulations [18]. Additionally, insights into data privacy stress the importance of robust data protection practices to prevent technology misuse and protect personal information, ensuring AI applications uphold individual rights and freedoms [19]. These principles, drawn from a broad array of previous research, constitute a comprehensive framework essential for the ethical deployment of AI technologies across all domains.

Serious concerns have been expressed about how AI might undermine the basis and essence of humanity [15]. Hypothetical syllogisms, which are arguments containing at least 1 hypothetical proposition as a premise, suggest a dystopian future with unfairness enacted by AI [16]. Recent studies also revealed that EDI concepts are not adequately implemented in the building of AI systems for health and oral health care [8,20-22]. For example, the use of AI in health care raises concerns about the privacy and security of sensitive medical information, particularly for patients who are vulnerable and may have a history of discrimination and mistrust in the health care system [23]. EDI is a conscious effort to include a diverse range of social identities and perspectives, including those typically marginalized in decision-making on social and health issues that impact their lives.

By definition, equity strives to treat everyone fairly with the recognition that each person has different circumstances and allocates the exact resources and opportunities needed to reach an equal outcome [20]. Diversity means including a range of people and perspectives such as those from different racial and ethnic backgrounds, genders, sexual orientations, ages, abilities, socioeconomic statuses, religions, and cultural backgrounds. Inclusion means the act of social inclusion of groups that are normally marginalized as well as the social values they subscribe to [20]. Paradoxically, some evidence suggests that AI can lead to fairness and efficiency by reducing the likelihood of human bias, but our decisions will determine how the future unfolds [16].

In this study, we argue that ethical and fair AI can be achieved only by integrating the EDI concept in the development and application of AI and machine learning models [15,16,20,24]. However, it is critically important that the building of AI systems be participatory in nature and includes diverse experts and stakeholders like system developers, health professionals, researchers, and policy makers, among other end users [2]. Unfortunately, this research shows that studies on the application of AI in health care have rarely included relevant stakeholders in the design, development, and implementation of AI systems [8,25,26].

The distinction between AI ethics and a framework concerning AI and EDI is important, emphasizing different facets of responsible AI development. AI ethics broadly encompasses moral values, guiding the development and application of AI technologies in a manner that respects human dignity and societal norms. It encompasses principles like transparency, accountability, and fairness, aiming to prevent AI systems from perpetuating harm or injustice [27]. In contrast, the framework focuses specifically on integrating these considerations as well as EDI principles throughout the AI system’s lifecycle—from design and development through deployment and monitoring. This lifecycle approach ensures that EDI principles are central, actively promoting inclusivity and mitigating biases [26]. This clarifies how AI ethics provides the foundational values, while the framework offers a structured approach to operationalize these values concretely and continuously across all stages of AI implementation.

Although studies have identified limitations, to the best of our knowledge, no studies to date have explored the feasibility and challenges of incorporating EDI variables, concepts, practices, and outcomes within AI technologies in the field of health and oral health care. There is a need to explore how EDI principles can be integrated into the lifecycle of AI technologies and gain an understanding of the relevant stakeholders’ perspectives on societal challenges and implementation of EDI in AI technologies for health care, oral health care, and related research. This study aimed to co-design the EDAI framework for integrating EDI throughout the lifecycle of AI in partnership with representatives of socio- or ethnocultural communities and end users, as well as with an international or intersectoral or interprofessional team of policy makers, researchers, clinicians, and industry representatives.

## Methods

### Study Design

Our research project began with a gap analysis and a comprehensive systematic scoping review, which explored the integration of EDI principles into the life cycle of AI within health and oral health care settings. The results of this initial systematic scoping review have been published separately [22]. In this paper, we focus on the other phases of the project, which is directly related to the framework development stage. This study followed a qualitative exploratory study design with a constructive grounded theory methodology approach and was presented according to the SRQR (Standards for Reporting Qualitative Research) guideline (Multimedia Appendix 1). Grounded theory enables “the generation of theories of process, sequence, and change pertaining to organizations, positions, and social interaction” [28]. This theory is a systematic methodology in which the researcher is not bound to select an existing theoretical framework. Instead, the findings may serve as the foundation for the development of new theories [29,30]. Therefore, for developing conceptual frameworks from diverse texts like the one presented in this study, the grounded theory technique is appropriate and insightful [31]. Constructivist grounded theory is a type of grounded theory that is based on constructivism [32]. It is centered on the coconstruction of experiences and meanings of the area of inquiry with the participants [32].

### Ethical Considerations

This study received ethics approval from the McGill University Institutional Ethics Committee (A03-E10-23A). Informed consent was obtained from all participants. Participants were also informed of their right to opt out at any time. Data collected in this study were deidentified; personal identifiers such as names were not recorded. Participants were not compensated monetarily; however, food was provided during the workshop.

### Study Phases

This research was conducted in 3 phases, and each phase is explained below in detail.

#### Phase 1: Gap Analysis

A comprehensive scoping review has been performed to explore what and how EDI principles and practices have been integrated into the conception, design, development, and implementation of AI studies conducted in health and oral health care settings [22]. The results of the scoping review indicate the lack of a comprehensive framework in health care, in which the principles of EDI are systematically deliberated and applied to the AI lifecycle [22]. Therefore, an expert group workshop was planned to develop a comprehensive framework, that is, EDAI, for health and oral health care settings. The review results informed the EDAI framework’s development.

#### Phase 2: Expert Group Workshops

##### Establishing a Multidisciplinary Team Involving Stakeholders and Collaborators

The framework development process was structured around 2 strategic workshops. The initial workshop in Montreal, Canada, centered on collaborative creation, bringing together diverse stakeholders to codevelop the framework’s foundational elements. Building on this groundwork, the second workshop in Geneva, Switzerland, was dedicated to refining and validating the preliminary version. This 2-phase approach ensured a comprehensive, iterative process that incorporated broad expertise while allowing for critical assessment and enhancement of the framework’s applicability. The purposeful sampling with maximum variation was used to enroll members from an intersectoral or multidisciplinary background (engineering, dentistry, medicine, AI systems, ethics in AI, communication, and social sciences) including researchers, clinicians, knowledge users, patient representatives, community partners, policy makers, public health agencies, and industry representatives. The selection of stakeholders started 1 year before the first workshop to build an expert research group. The team members were consciously selected to represent excellence in research activities or leadership and diversity in socio- or ethnocultural and geographical contexts. The team’s composition is in line with the participatory approach and the concept of codevelopment, which are at the core of this project. Most of the team members had a long-established working relationship and had successfully collaborated on various projects previously.

##### Data Collection

#### Overview

The study follows a qualitative research approach and includes 2-day, by-invitation, in-person workshops with focus group discussions to co-design the EDAI framework [21]. According to Ahmed and Mohd Asraf [33] and Lain [34], workshops are essential for prolonged engagement and are regarded as being one of the primary ways of establishing the credibility of the results of a qualitative study and upholding the trust between the researchers and participants. The workshops continued for 2 days and involved representatives from diverse backgrounds including government and nongovernment organizations, visible minority community leaders including Black communities, representatives from the World Health Organization, and the participation of health and oral health care providers, academia, patients, industry partners, and policy makers [21].

#### Workshop Engagement Exercises

The workshops included plenary presentations, round table working groups, and focused group discussions as a research methodology [21,35]. At the preworkshop phase, to facilitate the exchange of ideas, research team members prepared a draft document relevant to the area of research and distributed it to meeting participants 2-3 weeks before the workshops [21].

- Workshop presentations: Individuals were selected from the attendees to provide presentations on their previous experiences in EDI to enhance the discussions. A semistructured focus group discussion guide was developed before the workshop based on a review of the literature and previous work of team members [21]. A panel presentation linked the presentations to focus groups and included expert opinions on these questions: (1) What are the visions and values that guide the integration of EDI throughout the lifecycle of AI? (2) What are the facilitators and barriers to the integration of EDI throughout the lifecycle of AI within health and oral health care? (3) What should be the indicators of EDI in health and oral health care data and how can we measure the EDI variables throughout the lifecycle of AI? and (4) How a conceptual framework should be framed and what are the main elements?
- Round table working groups and focused group discussions: The presentation session was followed by focused group discussion groups based on qualitative methodology and inductive reasoning [21]. The discussion groups answered the same questions that had been asked of the panel participants.

Each focus group had a moderator and notetaker with 5-6 purposefully selected participants with maximum variation and lasted for 45-60 minutes. Afterward, there was a large group discussion among the workshop attendees to further explore the subject and reach a consensus together. All proceedings notes were written by the notetakers. All the notes and memos were compiled for data analysis.

#### Workshop Conclusion

The workshops were concluded with a summary of the workshop by restating the main points and identifying components for preliminary data analysis.

##### Data Analysis

Data analysis was followed immediately after each workshop. An inductive thematic analysis was done manually (SAR, EE, and RS). As per Braun and Clarke [36], the analysis began with the generation and collation of initial codes, which will then be sorted into multiple potential themes. These potential themes were then combined to form overarching key themes. During this process, working groups were created across team members to ensure data triangulation.

We followed the 8 phases of “conceptual framework analysis” within the grounded theory approach to systematically identify and interpret concepts, categories, and their relationships as explained by Jabareen [31]. Phase 1 is the mapping of data sources consisting of a literature review and workshop with focus group discussions [31]. Since EDI in AI is an unexplored concept, firsthand knowledge and experience using primary data collection was deemed important [37]. In phase 2, categorizing the selected data, and phase 3, identifying and naming concepts, the first set of concepts was inductively developed using line-by-line coding of focused group discussions [31,38]. After the identification of the patterns within the concepts, focused coding was initiated to establish initial categories [31,39]. Phase 4 is deconstructing and categorizing concepts, and phase 5 is integrating concepts and refining the coding by iterative assessment [31,37]. It included deconstructing and organizing the concepts and then integrating similar concepts together [31]. Constant comparative technique at each level of coding was used to assess similarities and differences for refining the concepts [32]. Phase 6, synthesis and resynthesis, included organizing the EDAI conceptual framework by inductively sorting the categories and grouping them under 3 main dimensions [31,37]. Phase 7, validation, and phase 8, rethinking the conceptual framework, were performed via triangulation, reflexivity, and participant feedback during the second workshop and via emails and digital meetings during data analysis [31,37].

#### Phase 3: Framework Development and Stakeholder Reviews

Based on the workshops’ key impressions and group discussions, the EDAI framework was developed and discussed with all participants electronically and in the second workshop. Their feedback was sought and iteratively incorporated to develop the final framework. Table 1 illustrates the key steps and the details of the process across the different phases of the study.

**Table 1:** Summary of study phases and steps.

Phase and step		Details
**Phase 1: gap analysis**		
	1. Scoping review	A comprehensive scoping review was performed to explore how EDI^a^ principles are integrated into AI^b^ studies in health and oral health care settings.
**Phase 2: expert group workshops**		
	2. Establishing a multidisciplinary team	A multidisciplinary team was built through purposeful sampling with maximum variation, including stakeholders from diverse backgrounds (eg, engineering, dentistry, medicine, AI, ethics, and social sciences). The selection began 1 year before the first workshop. The team composition emphasized codevelopment, participatory approaches, and diversity in socio- or ethnocultural and geographical contexts.
	3. Data collection	Both 2-day, in-person workshops involved focus group discussions to co-design the EDAI framework. Representatives from diverse backgrounds, including government, nongovernment organizations, minority communities, academia, and industry, participated. The workshop included plenary presentations, round table working groups, and focused group discussions, with preworkshop preparations such as draft documents distributed to participants. Focus groups addressed key questions on integrating EDI in the AI lifecycle, identifying facilitators and barriers, and defining EDI indicators and conceptual framework elements. Discussions were moderated, and notetakers documented all proceedings for data analysis.
	4. Workshop conclusions	The workshops concluded with a summary and identification of components for preliminary data analysis.
	5. Data analysis	Inductive thematic analysis was conducted manually. Initial codes were generated and sorted into potential themes, which were then refined and organized into key themes through collaborative efforts. The 8 phases of “conceptual framework analysis” (eg, mapping data sources, categorizing data, identifying concepts, and integrating concepts) were used. Constant comparative techniques were used to refine the coding, and the EDAI conceptual framework was organized into 3 main dimensions.
	6. Validation	Validation and refinement of the conceptual framework were achieved through triangulation, reflexivity, and participant feedback via emails and digital meetings as well as during the second workshop.
**Phase 3: framework development and stakeholder reviews**		
	7. Framework development	Based on workshop findings, the EDAI framework was developed and discussed with participants electronically. Feedback was iteratively incorporated to finalize the framework.

^a^EDI: equity, diversity, and inclusion.

^b^AI: artificial intelligence.

## Results

### Overview

We identified the knowledge gaps through a past systematic scoping review, the results of which were published in another paper (phase 1) [22]. The participants involved in phase 2 and phase 3 of the study included over 60 representatives from diverse backgrounds and expertise from government and nongovernment organizations, visible minority community leaders including Black communities, public health agencies, health and oral health care providers, AI and health services researchers, patients, industry partners, and policy makers. This diversity ensured balanced representation not only in terms of sex, gender, and expertise but also included other aspects such as race, ethnicity, socioeconomic background, age, and disability status.

We also included international participants and people from minority groups and marginalized populations. By actively recruiting from varied demographics, we aimed to address historical disparities and support equitable participation, thereby enriching the quality and impact of our research findings (phase 2 and phase 3). Five themes emerged from the data collected in phases 2 and 3. Table 2 illustrates the coding data structure of the collected data. This table illustrates initial, focused, and theoretical codes to classify the factors affecting EDI throughout the lifecycle of AI at micro (individual), meso (organization), and macro (system) levels to reach the final outcome of quadruple aims of health care by ensuring equitable, diversified, and inclusive care.

**Table 2:** Coding data structure.

Initial codes	Focused codes	Theoretical codes	Core category	Final outcome
Awareness about AIa and EDIb Inclusion of diverse perspectives and voices Addressing cultural sensitivities while respecting individual identities Incorporating principles of social justice and decolonization	Enhancing equitable access to health and oral health care Incorporating diverse perspective Inclusion of cultural safety and social justice	Role of EDI and AI in health and oral health care settings	Based on theoretical codes, classifying the factors affecting EDI throughout the lifecycle of AI (planning, data collection, designing, training, deployment, and decision-making) at micro (individual), meso (organization), and macro (system) levels	To achieve quadruple aims of health care by ensuring equitable, diversified, and inclusive care
Transparent use of data Trust, confidentiality, and privacy Political will	Responsible and ethical use of AI Mutual respect among stakeholders Supportive policy and regulation	Vision and values that will guide the integration of EDI in the life cycle of AI	Based on theoretical codes, classifying the factors affecting EDI throughout the lifecycle of AI (planning, data collection, designing, training, deployment, and decision-making) at micro (individual), meso (organization), and macro (system) levels	To achieve quadruple aims of health care by ensuring equitable, diversified, and inclusive care
Awareness about AI and EDI Access to health and oral health care Demographic factors	Factors affecting at the levels of patients, health care providers, AI developers, policy makers, and stakeholders	The facilitators and barriers to the integration of EDI Indicators of EDI in health and oral health care data	Based on theoretical codes, classifying the factors affecting EDI throughout the lifecycle of AI (planning, data collection, designing, training, deployment, and decision-making) at micro (individual), meso (organization), and macro (system) levels	To achieve quadruple aims of health care by ensuring equitable, diversified, and inclusive care

^a^EDI: equity, diversity, and inclusion.

^b^AI: artificial intelligence.

The current iteration of the EDAI framework is recognized as a preliminary version, designed to set the foundational elements necessary for integrating EDI principles within the AI lifecycle. Given the iterative nature of grounded theory, a second iteration of framework development is done during the second workshop. This session aimed to engage a broader sample and incorporate extensive data analysis and stakeholder input. The objective was to further refine and enhance the framework, thus improving its robustness and applicability in diverse health care settings.

### Theme 1: The Role of EDI and AI in Health and Oral Health Care Settings

Participants proposed leveraging AI to enhance access to health and oral health care, particularly in remote areas, by improving referral pathways. They emphasized that AI could address obstacles like cost and distance by offering alternative options such as telehealth. The discussions also highlighted AI’s potential in studying human behavior within health care and predicting health outcomes for diverse populations and the diverse use of AI in decision-making. They recommended organizing education, awareness, and training workshops to improve AI literacy among health care providers and the public, including marginalized communities. Furthermore, participants stressed the significance of incorporating EDI in patient data collection and analysis to reduce health and oral health disparities.

### Theme 2: Vision and Values That Will Guide the Integration of EDI in the Life Cycle of AI

During the discussions, participants stressed the importance of addressing biases and discrimination in AI algorithms and ensuring fair representation in the AI algorithms including the decision-making process. They emphasized the need to achieve this by including diverse perspectives and voices, empowering marginalized communities, and addressing cultural sensitivities while respecting individual identities. Additionally, the participants highlighted the significance of incorporating principles of social justice and decolonization at various stages of the AI life cycle, including problem definition, data collection, designing, training, deployment, and decision-making. Responsible and ethical use of AI was a key aspect that participants stressed throughout the conversations.

### Theme 3: The Facilitators and Barriers to the Integration of EDI Throughout the Lifecycle of AI Within Health and Oral Health Care

Table 3 depicts the facilitators and barriers to integrating the EDI within AI that were discussed within the workshop at the levels of patients, health care providers, AI developers, policy makers, and stakeholders. Participants stressed the importance of establishing an EDI committee for each stage of the AI lifecycle with a mandate to define fairness from an EDI perspective and ensure context-specific implementation.

**Table 3:** Facilitators and barriers to integration of EDIa in AIb.

Level and facilitators		Barriers
**At patients’ level**		
	Enhancing education and awareness regarding AI and EDI to alleviate patient resistance and fears. Ensuring clear and transparent communication with patients about the purpose and process of data collection for AI model development. Providing access and training of AI to patients.	Resistance to AI integration can arise due to fear and intimidation stemming from complex technological jargon, concerns about potential job loss, and negative portrayals in popular media. Marginalized communities may exhibit heightened apprehension toward AI. Limited technology training and lack of familiarity with AI applications, often exacerbated by low socioeconomic conditions. Generational disconnect among users and technology knowledge gap. Privacy concerns regarding patient consent.
**At health care providers’ level**		
	Implementation of training programs and knowledge enhancement initiatives for health care providers. Creation of ethical guidelines and policy frameworks concerning the collection and use of patient data.	Lack of EDI and AI literacy and understanding among health care providers.
**At AI developers’ level**		
	Fostering the inclusion of diverse perspectives in AI development and implementation and involving affected communities during the conceptualization stage—ensuring AI solutions address their specific needs and concerns. Identifying inclusive data and adopting participatory, community-oriented approaches in data collection—ensuring inclusivity and representation. Continuous feedback and evaluation of AI algorithms to correct biases and improve reliability. Adopting a transparent approach in the AI development stage to build trust and facilitate EDI integration. Asking the right questions focused on the problem rather than the technology can lead to more relevant and impactful AI solutions.	Limited knowledge and research on dimensions of EDI result in challenges in defining and categorizing diverse groups. Bias and discrimination embedded in human data and missing data can lead to biased AI predictions. Inadequate representation of diverse groups in the development process can lead to biased AI solutions. Misconduct in responsible research and data misuse. Limited resources for developing AI tools with integrated EDI can hinder progress.
**At policy makers’ level**		
	Having political will and financial support from policy makers can facilitate the identification of AI needs and the development of inclusive and equitable AI. Supportive policy and regulation frameworks for responsible and fair AI development and implementation.	Lack of policy frameworks around integration of EDI through the lifecycle of AI. The lack of regulatory authority and governance can create challenges in assessing AI integration from an EDI perspective, potentially leading to ethical and fairness concerns. Politicians exerting pressure about how society is using or will use AI, often for political gain. The current education system may limit diversity in AI developer groups.
**At all stakeholders’ level**		
	Connecting end users’ needs with stakeholders to facilitate EDI integration. AI literacy and educating stakeholders at all levels, including users, providers, and the community, about AI and its implications can build a better understanding and acceptance of AI solutions.	The lack of common vocabulary among different professionals, such as researchers, clinicians, AI developers, and consumers, can create obstacles in integrating EDI within the lifecycle of AI. AI’s potential to generate wealth may exacerbate societal disparities, with power dynamics among stakeholders influencing AI and EDI control. Privacy concerns related to data ownership.

^a^EDI: equity, diversity, and inclusion.

^b^AI: artificial intelligence.

### Theme 4: The Indicators of EDI in Health and Oral Health Care Data and Their Measurement

We identified the EDI indicators through a past scoping review, the results of which were published in another paper [22]. During the workshop, participants categorized the EDI indicators in health and oral health care into 3 levels: individual (micro level), community (meso level), and society (macro level). Table 4 details the identified EDI indicators under categorized levels.

**Table 4:** Refinement of the conceptual EDAI framework.

Levels and indicators		Details
**Micro level (individual level)**		
	Sociodemographic	Age, gender, residence, education level, occupation, income, marital status, socioeconomic status, race or ethnicity, religion, literacy, health literacy (including for artificial intelligence), cultural or traditional practices, immigration or acculturation, and family or peer or community-based influences
	Health and functional factors	Physical health, mental health, mobility, and cognitive function
	Psychobiological factors	Health beliefs, attitude, emotions, self-esteem, perceived health status, and genetics
	Behavioral	Lifestyle factors and habits (eg, tobacco and alcohol)
**Meso level (organization level)**		
	Health care system	Existing health system at the place of inquiry
	Education system	Exiting education system at the place of inquiry
	Community infrastructure	Physical infrastructure and rurality or remoteness
	Network and community support	Teamwork and shared ownership
**Macro level (system level)**		
	Colonialism	Political, socioeconomic, and cultural domination by foreign power
	Intersectionality	Racism, intersectional discrimination, and social exclusion
	Governance	Decision-making, accountability, and leadership (eg, health care structure and funding agency)
	Social belief	Social values and beliefs

To measure these EDI indicators and variables throughout the lifecycle of AI, participants stated that various tools and methods can be used including quantitative methodological analysis, surveys, interviews, anonymous self-reported questionnaires, image-based data collection, tracking location, and soliciting user feedback. Additionally, qualitative evaluation methods, such as reflective texts or live feedback from users, were mentioned as another source for providing valuable insights into EDI and the user experience with AI-enabled technologies and tools.

### Theme 5: Framing the EDAI Framework

**Figure 1:**
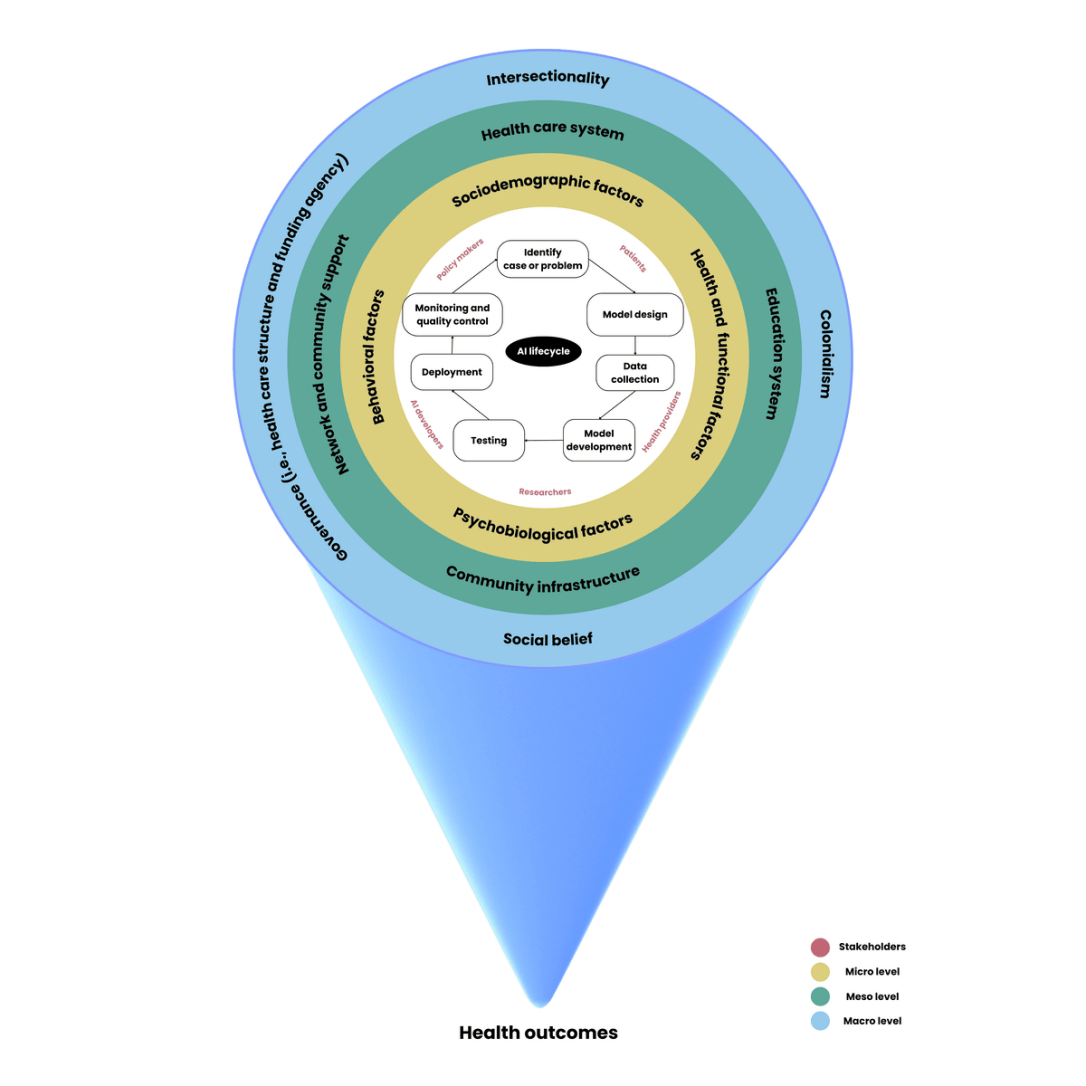
The proposed EDAI framework. AI: artificial intelligence.

Integration of EDI in AI follows a multifactorial approach. This approach is shown in Figure 1. AI-based health and oral health practices should underpin all relevant factors at micro, meso, and macro levels. Therefore, the final framework is structured over these 3 levels: micro (individual level), meso (organization level), and macro levels (system level) with the indicators underpinning the whole lifecycle of AI. The micro level comprises smaller entities like individuals or families and their interactions. The meso level involves groups or communities or organizations and their interactions. The macro level encompasses broader systems such as policies or government structures at institutional, national, or international levels and their interactions. These 3 levels are interconnected and interrelated and significantly influence each other.

At the micro level, EDI encourages diverse representation and involvement of people of all sociodemographic factors (ie, age, gender, residence, socioeconomic status, and marital status) who may have diverse capabilities, skills, expertise, and values. It inspires people from various sociocultural factors such as race or ethnicity, religion, health literacy, cultural or traditional practices, immigration or acculturation, and family or peer or community-based influences. Moreover, respecting cultural diversities can widen the scope of AI-based health care. Integration of EDI within AI can positively impact an individual’s psychobiological factors including their health beliefs, attitude, emotions, self-esteem, and perceived health status. Furthermore, EDI-informed approaches are helpful in inculcating healthy lifestyle behaviors and habits by fostering an inclusive environment that respects and values diverse cultural perspectives.

At the meso level, health systems comprising both public and private health organizations and institutes should foster the integration of EDI within the lifecycle of AI in health and oral health care by emphasizing on the provision of a culturally safe environment for patients and care providers and ensuring the presence of proficient professionals. The education system should ensure that all in the school community—students, their families, teachers, staff, and administrators—feel valued and heard. Community infrastructure needs to be considered while working on AI-EDI to have better representation of all community members irrespective of physical location, rurality or remoteness, representativity, and accessibility. Furthermore, networks and community support such as social networks, community groups, and peer support should also focus on diverse representation with teamwork and shared ownership.

At the macro level, focusing on intersectionality can aid in understanding the complex interactions of various factors at micro and meso levels, thereby striving to achieve social justice. Integration of EDI within the lifecycle of AI should emphasize decolonization by minimizing the impact of settler colonialism, racism, and discrimination and acknowledging the historical injustices and systemic inequalities that have impacted marginalized communities. Furthermore, social beliefs play a significant role in shaping attitudes and actions toward EDI since positive social beliefs can foster environments where individuals from all backgrounds feel valued and empowered. In the context of EDI in the AI lifecycle, governance plays a critical role in setting policies, implementing practices, and enforcing regulations that promote EDI. Finally, in our workshops, participants also emphasized the importance of responsible AI including reliability and safety, privacy and security, transparency, and accountability [40], which should be considered during development and implementation.

## Discussion

### Principal Findings

Our findings reveal significant gaps in EDI considerations at various stages of the AI lifecycle, from problem definition and data collection to implementation. In response, this study developed a guiding framework—the co-designed EDAI framework—that integrates EDI principles and indicators comprehensively throughout the AI lifecycle. The field of AI ethics, which emerged alongside the evolution of AI technologies, has grown in importance, as AI systems have become more advanced. Focused initially on issues like algorithmic bias, transparency, privacy, and fairness, AI ethics seeks to align AI technologies with moral principles and values. However, it is distinct from EDI in AI, which extends beyond ethical considerations to emphasize the integration of EDI throughout the AI lifecycle. Consideration of EDI not only aims to prevent biases but also ensures diverse representation and targets social equity and inclusivity.

Despite the advantages of AI, there are some concerns that underscore the necessity of consciously integrating EDI principles into the lifecycle of AI systems to ensure they are socially responsible and equitable. These concerns include discrimination, unfair treatment, and risks of developing or intensifying health-related inequalities [41]. Recent systematic reviews of EDI in AI found a lack of EDI considerations in the dataset and AI-based academic literature and a lack of EDI principles and indicators to be integrated into the AI lifecycle [22,41]. The studies also highlighted the need to integrate EDI and racial justice principles and practices in AI; however, there is no guideline or framework to promote this integration. This study is addressing this knowledge gap by identifying EDI principles and indicators to be integrated into the AI lifecycle and developing a guiding framework to guide the next-generation AI system development and implementation.

Our results highlight the importance of an EDI-sensitive approach in the life cycle of AI, reducing inequities and inequalities in health and oral health. The EDI concepts should be integrated not only during AI system development but also throughout its lifecycle, including AI planning, data collection, designing, training, deployment, and decision-making. Moreover, the study results emphasize the fair representation of all stakeholders including patients, health care providers, AI developers, and policy makers. Therefore, we developed an EDAI framework in this study encompassing several EDI indicators in health and oral health care data at the individual (micro), community (meso), and society (macro) levels underpinning the whole lifecycle of AI.

In other studies, the AI or Machine Learning Consortium to Advance Health Equity and the Researcher Diversity Ethics and Equity Workgroup has established ethics and equity principles. These principles focus on fostering trust with communities, intentionally designing and implementing AI systems, creating solutions, building capacity, and re-evaluating institutional mechanisms to reset the rules [42]. The group has also developed a glossary containing 12 terms, with particular emphasis on AI development in health equity research [42]. Furthermore, with the help of the Delphi method, Rokhshad et al [43] developed a checklist of 11 ethical principles specific to AI-based dentistry including equity and diversity as major principles. However, these studies are providing worthy groundwork by focusing on ethical principles and health equity, the current studies lack identifying and integrating EDI principles and indicators in the AI lifecycle. The proposed EDAI framework offers a structured approach to exploring EDI concepts and the factors influencing them throughout the AI lifecycle. It addresses the need to identify and integrate EDI principles and indicators within AI systems.

A recent scoping review conducted by Singhal et al [44] emphasized the crucial integration of fairness, accountability, transparency, and ethics across all phases of AI system development from conceptualization to deployment. The review also suggests using diverse datasets to ensure a more accurate reflection of the world’s population [44]. Additionally, they advocate the active involvement of all stakeholders at every stage. Likewise, the EDAI framework outlined in this paper aims to address these recommendations, with particular attention to the factors influencing AI-driven health care technology across all stakeholder groups and throughout the lifecycle of AI system. In this line, Dankwa-Mullan et al [26] have also proposed a framework centered on integrating health equity and racial justice principles within each stage of the AI lifecycle to ensure ethical and moral behavior in AI system development. Similar to the proposed EDAI framework, various AI ethics frameworks have emphasized interdisciplinary stakeholders’ collaboration as one of the foundations of ethical and responsible AI [45,46]. This multifaceted interdisciplinary stakeholders’ collaboration can involve active participation from policy makers, health care providers, AI experts, and patients in AI-based ethical decision-making in health and oral health care [45,46].

A recent review of AI guidelines related to EDI by Cachat-Rosset and Klarsfeld [41] analyzed 46 published guidelines pinpointing 14 relevant principles and 18 practices. They reported fairness, justice, and nondiscrimination in a limited compliance approach within these guidelines. Their findings revealed a partial adherence to fairness, justice, and nondiscrimination within these guidelines, suggesting a constrained approach to compliance [41]. The authors recommended the need for holistic guidelines over the more technical ones [41]. Considering these recommendations, the EDAI framework endeavors to create a holistic structure that takes into account multiple factors at the individual, organizational, and societal levels.

EDI can be challenging to measure because definitions of variables, for example, gender may evolve, reflecting societal changes. Additionally, the measurement of EDI may vary depending on the specific context in which AI models are applied, making it challenging to establish a standardized set of indicators. Furthermore, fairness is context-dependent and relies on data collection methods, which can vary based on factors like self-identification of the data versus government documents. However, having a ready-to-use tool can help all the stakeholders assess the integration of EDI in health or oral health-based AI systems. Therefore, based on the findings of this study, the authors plan to create and validate an evaluation-based checklist and guidelines for the integration of EDAI in health and oral health care.

The developed framework can help AI developers, who are integral to the model, by increasing their awareness of EDI principles, thereby reducing inherent biases and ensuring that development teams are diverse and inclusive, leading to more equitable and effective AI solutions in health care. For instance, the EDAI framework could facilitate the development of AI-driven diagnostic tools that better account for demographic variations, ensuring that diagnoses are accurate across diverse populations. This not only improves patient outcomes but also addresses historical biases in medical research and treatment. Another example is health care management in which AI systems designed and implemented under the EDAI framework can optimize resource allocation and patient scheduling to prioritize accessibility and minimize disparities in care delivery. Furthermore, this framework can guide the development of AI tools for patient education and engagement, ensuring that materials are culturally relevant and accessible to all, thereby improving health literacy and patient autonomy across diverse communities.

### Strengths and Limitations

One of the strengths of this research work is its systematic approach and the intersectoral and multidisciplinary team. The team members were consciously selected to represent excellence in research activities and leadership and diversity in socio- or ethnocultural and geographical contexts. The team’s composition is in line with the participatory approach and the concept of codevelopment, which are at the core of this project. Moreover, the EDAI framework also attempts to understand the linkages among various indicators at individual, organizational, and system levels. However, this study has a few limitations. First, while we were unable to record the workshop sessions due to logistical constraints, detailed notes were diligently taken by designated notetakers to ensure the accuracy and richness of the data. Second, despite our efforts, we were not able to engage First Nations and Aboriginal people in this initial phase. However, we did include a diverse population from different communities, ensuring a wide range of perspectives, and we are committed to incorporating First Nations and Aboriginal voices in future iterations of the framework.

### Conclusions

Recent studies have found that there are significant gaps in EDI considerations within the AI lifecycle, from the problem definition and data collection stage to implementation. However, there is currently no existing guideline or framework to support this integration. This study aimed to address this gap by identifying EDI principles and indicators to be integrated into the AI lifecycle and developing a guiding framework to steer the development and implementation of future AI systems. The co-designed EDAI framework is comprehensive, encompassing a detailed compilation of EDI indicators across the AI lifecycle, spanning from individual to organizational and system levels for all stakeholders. These insights have the potential to reshape perspectives on integrating EDI into AI and offering valuable guidance to policy makers, service providers, and AI developers to implement change in AI practices.

## Appendix

Multimedia Appendix 1 SRQR (Standards for Reporting Qualitative Research) checklist.

## Abbreviations

AI: artificial intelligence
EDI: equity, diversity, and inclusion
SRQR: Standards for Reporting Qualitative Research

## Data Availability

The datasets generated and analyzed during this study are available from the corresponding author on reasonable request.
